# Skin snips have no role in programmatic evaluations for onchocerciasis elimination: a reply to Bottomley et al.

**DOI:** 10.1186/s13071-017-2090-z

**Published:** 2017-03-23

**Authors:** Mark L. Eberhard, Ed W. Cupp, Charles R. Katholi, Frank O. Richards, Thomas R. Unnasch

**Affiliations:** 1 Division of Parasitic Diseases and Malaria, Center for Disease Control and Prevention, Atlanta, GA 30329 USA; 2 Department of Entomology and Plant Pathology, Auburn University, Auburn, AL 36849 USA; 3 Department of Biostatistics, The University of Alabama at Birmingham, Birmingham, AL 35294 USA; 4 The Carter Center, 1 Copenhill, 453 Freedom Parkway NE, Atlanta, GA 30307 USA; 5 Department of Global Health, University of South Florida, Tampa, FL 33620 USA

**Keywords:** Skin snips, Onchocerciasis, Program evaluation

## Abstract

A critique of the recommendation that skin snips be used for post-MDA surveillance of formerly endemic onchocerciasis areas is provided. After considering several fundamental aspects of the use of this methodology within the context of prolonged mass distribution of ivermectin, we argue that skin-snipping has no value for monitoring onchocerciasis elimination programs.

## Letter to the Editor

We read with interest the recent article by Bottomley et al. [1] and were surprised that the authors concluded that skin snips have utilitarian value in evaluating the efficacy of onchocerciasis elimination programs three years after final mass drug administration (MDA). We strongly disagree that skin snips would have any practical utility in this or any other operational scenario in assessing whether to stop MDA or in post MDA transmission elimination monitoring. We discuss here four key concerns that were not addressed in the paper that led the authors to this erroneous conclusion.

**1. Conflicting assumptions in the use of the over dispersed distribution model.** The entire paper rests on the single key assumption that the over dispersed model is an appropriate representation for the number of microfilariae to be found in skin snips. A Poisson model is first put forward by the authors: *“If microfilariae are randomly distributed in the skin, then we can use the Poisson distribution to calculate the sensitivity directly from the density of microfilariae.”* There is no valid reason for this assumption except possibly for the ease of using mathematical derivations that are familiar to the authors. Later, the authors change their assertion,*“However, microfilariae are unlikely to be randomly distributed in the skin (e.g., the density may depend on the distribution of fertile worms in the body). If the distribution is non-random because microfilariae occur in “clumps”, then this will affect the sensitivity of skin snips because the chance that a snip contains no microfilariae is increased. To allow for this, we can use the negative binomial model rather than the Poisson to model the distribution of the microfilariae in the skin”.* The replacement of the Poisson model by the negative binomial model is generally done because it is believed that the data is subject to over dispersion. A critical observation made by Bottomley et al. [1] is that the chances that a skin snip contains no microfilariae are increased. It would seem reasonable that as an elimination program proceeds and the number of microfilariae decreases, that large numbers of zero snips will be detected in persons actually infected (and infectious to flies; see, for example, Davies et al. [2]).

Another issue not discussed but germane to the authors’ assumptions is that of the “zero inflation problem” that occurs as the number of microfilarial positive individuals in a community decreases. If one assumes that microfilariae are not uniformly distributed in the skin, there is the possibility that none are found due to where the snip is taken even though the person is infected or, alternatively, a snip is negative because the person is actually not infected. To compensate for this, additional statistical measures are required, i.e., using a mixture distribution of the Poisson/Negative Binomial and a “point” random variable at zero [3, 4].

**2. Lack of sensitivity.** Skin-snipping is a relatively insensitive method for use following long-term MDA. Indeed, when compared for accuracy (as evaluated by a more highly sensitive method, e.g., PCR), Thiele et al. [5] noted that skin snip microscopy demonstrated an insufficient sensitivity for reliable programmatic monitoring. This concept was addressed in the most recent World Health Organization (WHO) Verification Guidelines [6] that concluded that skin snip sensitivity was about 20% in populations with low disease prevalence. The Guidelines concluded that “Parasitological evaluation by skin snip microscopy … can be used to monitor progress during the first (treatment) phase of onchocerciasis elimination programmes, but **not to verify** (bold added) elimination”.

**3. Community-wide resistance to skin-snipping.** Skin-snipping is often painful and not well tolerated by a sizeable segment of the target population. Bottomley et al. [1] cite three papers in which population sampling was complicated by rejection of skin-snipping, yet the authors fail to mention this in their paper. For example, Ozoh et al. [7] stated that *“… the procedure [skin-snipping] is invasive and may cause secondary bacterial or viral infections. Even rural people are well informed about the dangers of human immunodeficiency virus transmission resulting from invasive procedures and are now likely to reject skin snipping.”* Resurrecting its use after a 3–5 year hiatus of non-treatment would most likely be unacceptable on a broad front. This phenomenon has already occurred repeatedly in several large community-wide studies and is documented in two more papers cited by the authors in which high refusal rates of participants available for skin-snipping were common place. In the large Mali/Senegal study, 40% of participants did not participate 20–22 months after the last Mectizan treatment, i.e., 22% were absent from the village at the time of examination and 28% refused to be examined [8]. In at least six cases, entire villages refused to participate in post MDA surveillance activities involving skin snips [9]. Further, given these precedents, to double the number of snips per person (four instead of two) in order to increase the sensitivity of the model (as recommended by Bottomley et al. [1]) would be a non-starter in virtually every MDA post-surveillance setting.

**4. Sample sizes required for statistical confidence obviate the usefulness of skin-snipping.** To state with 95% confidence that an infection rate is below 1%, roughly 300 people need to be examined and all must be found to be negative. However, this assumes the assay being used has 100% sensitivity. To a first approximation, the sensitivity of the assay is inversely proportional to the number that need to be screened. So if the skin snip assay is assigned a 20% sensitivity, the need to sample 300 people at 100% sensitivity translates to needing to examine 1500 people. Most onchocerciasis endemic communities targeted for MDA are not that large, which means in many cases everyone in each village would have to be snipped. As noted above, broad refusals would likely prevent this from being realized. Furthermore, even if it were possible to snip everyone in the community, an assay with a sensitivity of 20% would yield a false negative result 80% of the time. Simple probability calculations reveal that if it were possible to sample everyone in a community of 1000 individuals with a skin snip assay with a 20% sensitivity and all were found to be negative one could still not determine with 95% confidence that the actual prevalence in the community was 1% or less. This is because if there were 10 positive individuals in a community of 1000 people, the skin-snip probability that all these would be falsely declared negative is greater than 10%. Statistical confidence would be further skewed if we assume that persons refusing skin snipping are more likely to have refused MDA (increasing the likelihood that they would be mf positive).

In conclusions, there are several places where skin snips might be useful on a limited basis. One is during a drug trial where demonstration of direct action against microfilariae may be helpful. Another is for PCR confirmation of a suspected patent infection in a person who is Ov16 positive, as noted in the WHO Guidelines. Regardless, given these core mitigating factors noted above, we believe that Bottomley et al. [1] erred in calling for the use of skin-snipping as a method to determine whether to stop MDA and as a post-MDA surveillance procedure. The 2016 WHO Guidelines for the verification of elimination of onchocerciasis state emphatically that the use of skin snips is to be avoided as a means to determine whether or not to stop MDA, and to monitor post-MDA status as a lead up to verification of interruption of transmission. Entomological assessments, supplemented with serological testing, as currently recommended by WHO, is by far the best available approach for informing these critical decisions. Country program managers must be made aware of the extreme lack of usefulness of skin snips in assessing the elimination of *Onchocerca volvulus*.

## Abbreviations

MDA: Mass drug administration
WHO: World Health Organization
